# Histopathological and Molecular Data on *Myxobolus* spp. Infecting Bryconid Fish From the Brazilian Pantanal Wetland

**DOI:** 10.1111/jfd.70237

**Published:** 2026-07-09

**Authors:** Caroline Munhoz Meira, Milena Lucia Cornejo, Sarah Arana, Edson Aparecido Adriano, Antonio Augusto Mendes Maia

**Affiliations:** ^1^ Departamento de Medicina Veterinária, Faculdade de Zootecnia e Engenharia de Alimentos Universidade de São Paulo (USP) Pirassununga Brazil; ^2^ Instituto de Biologia Universidade Estadual de Campinas – UNICAMP Campinas Brazil; ^3^ Instituto de Ciências Ambientais, Químicas e Farmacêuticas Universidade Federal de São Paulo Diadema Brazil; ^4^ Departamento de Biologia Animal, Programa de Pós‐Graduação Em Biologia Animal, Instituto de Biologia Universidade Estadual de Campinas – UNICAMP Campinas Brazil

**Keywords:** histopathology, *Myxobolus pantanalis*, *Myxobolus salminus*, *Myxobolus umidus*, myxozoa, phylogeny, SSU rDNA

## Abstract

Previous studies described *Myxobolus umidus* infecting the spleen of *Brycon hilarii* , whereas *Myxobolus pantanalis* and *Myxobolus salminus* infecting the gill filaments of *Salminus brasiliensis* . In this study, we provided supplementary morphological and molecular data on these three *Myxobolus* species and histopathological analyses of the tissues infected by *M. umidus* and *M. pantanalis* in bryconid fishes caught from the Brazilian Pantanal wetland. A total of four *B. hilarii* and eleven *S. brasiliensis* specimens were examined. Among the four *B. hilarii* , one specimen exhibited several *M. umidus* plasmodia surrounded by a thin capsule of connective tissue in the spleen, but no inflammatory process was noted. Whereas two of the eleven *S. brasiliensis* individuals presented plasmodia of *M. pantanalis*. Fish infected with *M. pantanalis* were characterized by pronounced inflammatory reaction, blood congestion, hyperplasia of lamellar epithelium, lamellar fusion, and focal necrosis of chondrocytes in the gill filaments. *Myxobolus* species confirmation was achieved through morphological assessment and small subunit ribosomal DNA (SSU rDNA) sequencing performed on myxospores isolated from a single plasmodium of each species (*M. umidus, M. pantanalis,* and *M. salminus*). Phylogenetic analysis based on the newly generated SSU rDNA sequence of *M. salminus*, placed this species as sister to *Henneguya rotunda*, reinforcing the strong tendency for *Myxobolus* and *Henneguya* species to cluster according to the phylogenetic relationships of their fish hosts.

## 1 Introduction

*Brycon hilarii* Valenciennes, 1850, colloquially known as the Piraputanga, and *Salminus brasiliensis* (Cuvier, 1816), popularly known as the Dourado (Portuguese) and Jaw characin (English) are fish of the family Bryconidae (Froese and Pauly 2026; Gimênes Junior and Rech 2022; Resende 2003; Vitti et al. 2013). *Salminus brasiliensis* is the largest bryconid and one of the biggest fish predators found throughout the La Plata Basin, playing an important ecological role regulating the populations of other fish and structuring the prey community. The species occupy a prominent position in commercial and recreational fisheries. *Brycon hilarii* is restricted to the Paraguay River sub‐basin and is highly valued for both human consumption and recreational fishing. Both species have played an important role in Brazilian commercial aquaculture (Flora et al. 2010; Gimênes Junior and Rech 2022; Vitti et al. 2013; Zaniboni Filho et al. 2006, 2017). Due to overfishing, river fragmentation by dams, and habitat degradation, *S. brasiliensis* has experienced population declines in parts of its distribution range, leading to fishing restrictions in some regions, including parts of the Brazilian wetland Pantanal (IMASUL 2024; Ribolli et al. 2021).

Thus, the importance of these bryconid fishes to the Pantanal rivers ecosystem, as well as the economic relevance for fisheries and aquaculture in South America underscores the importance of understanding the imbalance in the parasite–host interaction, when the infection potentially causes damage to these fish species, as is the case of Myxozoa, a highly diverse lineage of cnidarian endoparasites (Okamura et al. 2015). Investigating myxozoan infections in these species provides valuable insights into parasite diversity and their potential health impacts on wild and cultured fish populations. To date, twelve myxozoan species have been described infecting *B. hilarii* and *S. brasiliensis* , eight of which were collected from the Brazilian Pantanal wetland (Adriano et al. 2009; Azevedo et al. 2011; Carriero et al. 2013; Meira et al. 2025; Milanin et al. 2010). Among these, *Myxobolus umidus* Carriero et al. 2013, and *Myxobolus pantanalis* Carriero et al. 2013, parasites of the spleen of *B. hilarii* and the gill filaments of *S. brasiliensis* , respectively, have been studied only from a taxonomic perspective, lacking histopathological data. Additionally, *Myxobolus salminus* Adriano et al. 2009, a parasite of the gill filaments of *S. brasiliensis* , was described based on the morphology of the myxospores (light and electronic microscopy) and histopathological observations, lacking molecular data from the small subunit ribosomal DNA (SSU rDNA).

Thus, aiming to expand the understanding of these species and to fill the data gaps highlighted above, we provide complementary histopathological information for *M. umidus* and *M. pantanalis*, as well as SSU rDNA data and phylogenetic analysis for *M. salminus*, obtained from their hosts in the Brazilian Pantanal wetland.

## 2 Materials and Methods

Eleven *S. brasiliensis* specimens and four *B. hilarii* specimens were collected from the Miranda River, in the Pantanal wetland (municipality of Corumbá, Mato Grosso State, Brazil—19°24′52.5″S 57°22′44.3″W [distance variation of 3 km]) in September 2022. Collections were conducted under authorization from the Brazilian Ministry of the Environment (SISBIO n° 67,616 and SisGen n° A33CB83). Fish were captured using a gill net, transported alive to a makeshift field laboratory near the sampling sites, and maintained under continuous oxygenation in the same water from the collection environment. Euthanasia was performed through spinal cord transection, followed by biometric recording (weight and length) and external inspection for lesions/cysts. The study protocol was approved by CEUA/FZEA‐USP (Authorization No. 9465020224, ID 002267). Necropsy was subsequently conducted, exposing gills and the visceral cavity to detect organ alterations, myxozoan plasmodia, and myxospores. Host tissues containing parasites were fixed in absolute ethanol for molecular analysis and in 10% neutral‐buffered formalin for morphological and histopathological analyses.

Myxospores of *M. umidus*, *M. pantanalis*, and *M. salminus*, preserved in 10% neutral‐buffered formalin, were examined and photographed using a Leica DM500 light microscope equipped with a Leica ICC50 W4841 camera. Myxospores measurements were performed using Laz Ez software (v. 3.4.0), following the criteria established by Lom and Arthur (1989). For histological analysis, host tissues containing plasmodia of *M. umidus* and *M. pantanalis* fixed in 10% buffered formalin were processed via ascending ethanol dehydration (70%, 90%, 100%) and xylene clearing, embedded in paraffin, sectioned at 4–6 μm, and stained with Haematoxylin–Eosin.

Total genomic DNA was extracted from myxospores recovered from plasmodia in the gill and spleen and preserved in absolute ethanol using the *DNeasy Blood & Tissue Kit* (Qiagen, Hilden, Germany), following the manufacturer's protocol. DNA concentration and purity were measured using a NanoDrop 2000 spectrophotometer (Thermo Fisher Scientific Inc., Waltham, MA, USA) at 260 nm. Amplification of the SSU rDNA was performed by polymerase chain reaction (PCR) following Mathews et al. (2020), using a ProFlex PCR System thermal cycler (Applied Biosystems, Foster City, CA, USA). Two primer sets were used for the three *Myxobolus* spp.: ERIB1 (Barta et al. 1997) with ACT1r (Hallett and Diamant 2001), and Myxgen4F (Diamant et al. 2004) with ERIB10 (Barta et al. 1997), generating amplicons of approximately 1000 bp and 1100 bp, respectively. The PCR thermal profile consisted of an initial denaturation at 95°C for 2 min, followed by 35 cycles of denaturation at 95°C for 60 s, annealing at 58°C–60°C (ERIB1–ACT1r) or 58°C (Myxgen4F–ERIB10) for 60 s, and extension at 72°C for 90 s. A final extension step was performed at 72°C for 5 min. PCR products were visualized on a 1.5% agarose gel TAE buffer (Tris 40 mM, acetic acid 20 mM, EDTA 1 mM) stained with SYBR Safe DNA Gel Stain (Thermo Fisher Scientific Inc., Waltham, MA, USA), under an LED transilluminator (Kasvi, São José dos Pinhais, PR, Brazil). Fragment sizes were estimated against the 1 Kb Plus DNA Ladder (Thermo Fisher Scientific Inc., Waltham, MA, USA). Amplicons were purified using the *QIAquick PCR Purification Kit* (Qiagen, Hilden, Germany). Sequencing was performed on an ABI 3730 DNA Analyser (Applied Biosystems, Foster City, CA, USA) with the *BigDye Terminator v3.1 Cycle Sequencing Kit* (Applied Biosystems), employing the same PCR primers and two internal primers, MC5 and MC3 (Molnár et al. 2002), to obtain overlapping fragments. Sequences were edited and aligned with BioEdit v7.1.3.0 (Hall 1999). A nucleotide BLAST search was conducted to determine sequence similarity with GenBank references and to assist in the identification of species previously described in the literature based on SSU rDNA data (Altschul et al. 1997).

Phylogenetic analysis was performed using SSU rDNA sequences that showed the highest BLASTn's similarity regarding the sequence of *Myxobolus salminus* here obtained. Only sequences exhibiting at least 85% similarity were selected for inclusion. Additionally, SSU rDNA sequences from other South American *Henneguya* and *Myxobolus* species were incorporated to provide a broader phylogenetic context. Sequence alignment was done using the ClustalW algorithm (Thompson et al. 1994) through the KEGG GenomeNet platform (Kyoto University Bioinformatics Center, n.d.). Maximum likelihood (ML) analysis was conducted on the PhyML 3.0 online server (Guindon et al. 2010), and the best‐fit nucleotide substitution model (GTR + *R*) was selected using the Smart Model Selection in PhyML (Lefort et al. 2017), based on the Akaike Information Criterion. Node support was assessed with 1000 bootstrap replicates. The resulting consensus tree was visualized and edited using FigTree v1.4.4–1 (Rambaut 2018). *Ceratomyxa amazonensis* (KX236169), and *Ceratomyxa vermiformis* (KX278420) were used as outgroup taxa. To evaluate genetic distances between myxozoan species clustering with the *M. salminus* sequence, pairwise comparisons using the p‐distance model were performed in MEGA11 (Tamura et al. 2021).

## 3 Results

Among the four *B. hilarii* specimens examined, one (25%) exhibited splenic plasmodia containing myxospores compatible with those of *M. umidus* (Figure 1A). In *S. brasiliensis* , three (27.2%) of the eleven specimens presented plasmodia harbouring myxospores consistent with species of the *Myxobolus* genus in the gill filaments. Further morphological analyses revealed that these myxospores belonged to two distinct species, with two *S. brasiliensis* specimens infected by *M. pantanalis* (18.2%) and one by *M. salminus* (9.1%) (Figure 1B,C) (Table 1).

**FIGURE 1:**
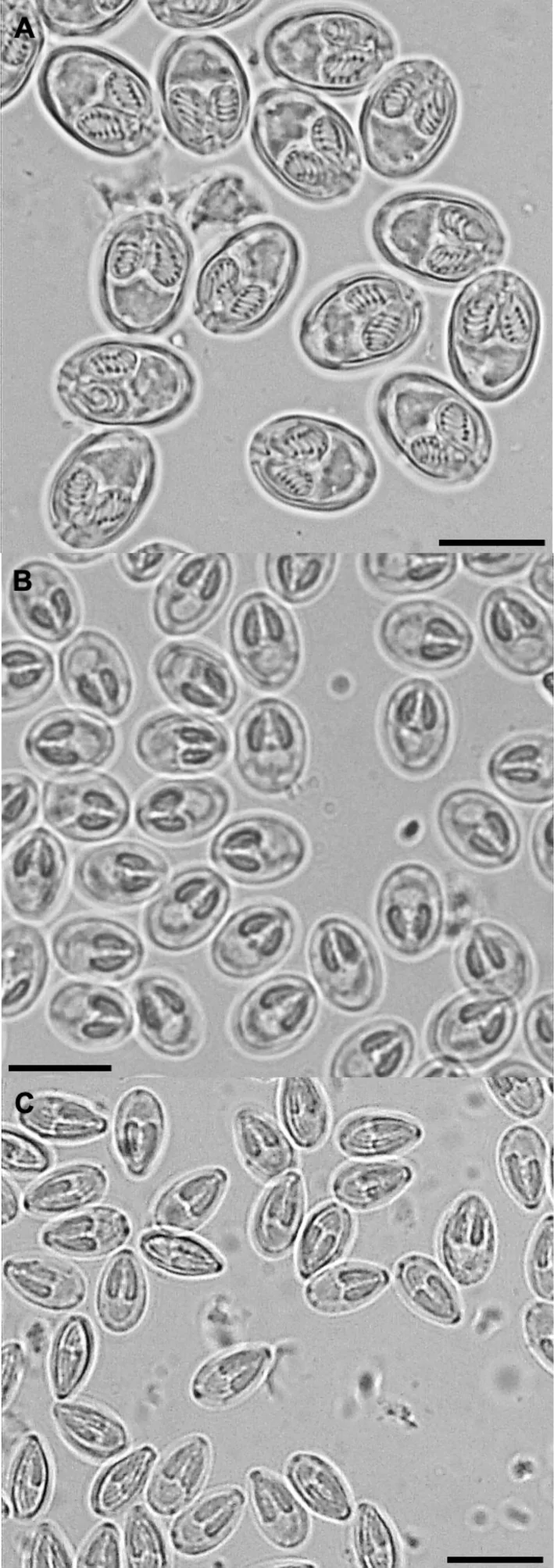
Photomicrographs of *Myxobolus* spp. infecting bryconid fishes. (A) Mature myxospores of *Myxobolus umidus* from the spleen of *Brycon hilarii* . (B, C) Mature myxospores of *Myxobolus pantanalis* and *M. salminus* from the gill filaments of *Salminus brasiliensis* . Scale bars = 10 μm.

**TABLE 1:** Data of *Myxobolus umidus*, *Myxobolus pantanalis*, an*d Myxobolus salminus par*asites of bryconids fish of this study.

Species	Spore length	Spore width	Thickness	NL	NW	NCNT	Site of infection	Host
*Myxobolus umidus*	13.1 ± 0.5	8.7 ± 0.5	7.4 ± 0.3	5.9 ± 0.5	3.4 ± 0.5	5	Spleen	*Brycon hilarii*
*Myxobolus pantanalis*	9.2 ± 0.3	6.6 ± 0.3	3.8 ± 0.2	4.1 ± 0.2	1.8 ± 0.2	—	Gill filaments	*Salminus brasiliensis*
*Myxobolus salminus*	10.7 ± 0.3	6.3 ± 0.3	4.7 ± 0.3	5.6 ± 0.3	1.5 ± 0.2	—	Gill filaments	*Salminus brasiliensis*

*Note:* Myxospore dimensions, infection sites, and host are given. All measurements are ranges in μm and/or means ± SD.

Abbreviations: −, No data; NCNT, Number of coils of nematocysts tubules; NL, Length of nematocysts; NW, Width of nematocysts.

Histopathological analysis of the spleen of *B. hilarii* infected by *M. umidus* revealed the presence of several large spheric plasmodia, with up to 0.8 mm within the splenic parenchyma (Figure 2A). These plasmodia were surrounded by a delicate connective tissue capsule formed by a layer of elongated cells in an epithelioid arrangement (Figure 2B,C). The splenic parenchyma appears preserved, and no inflammatory reaction was observed. No free myxospores were observed in the white or red pulp of the spleen, including inside of melanomacrophage centers (Figure 2C,D). In the periphery of the plasmodia, it was possible to note a layer of sporogonic developmental stages, whereas mature myxospores occupied deeper regions (Figure 2B–D).

**FIGURE 2:**
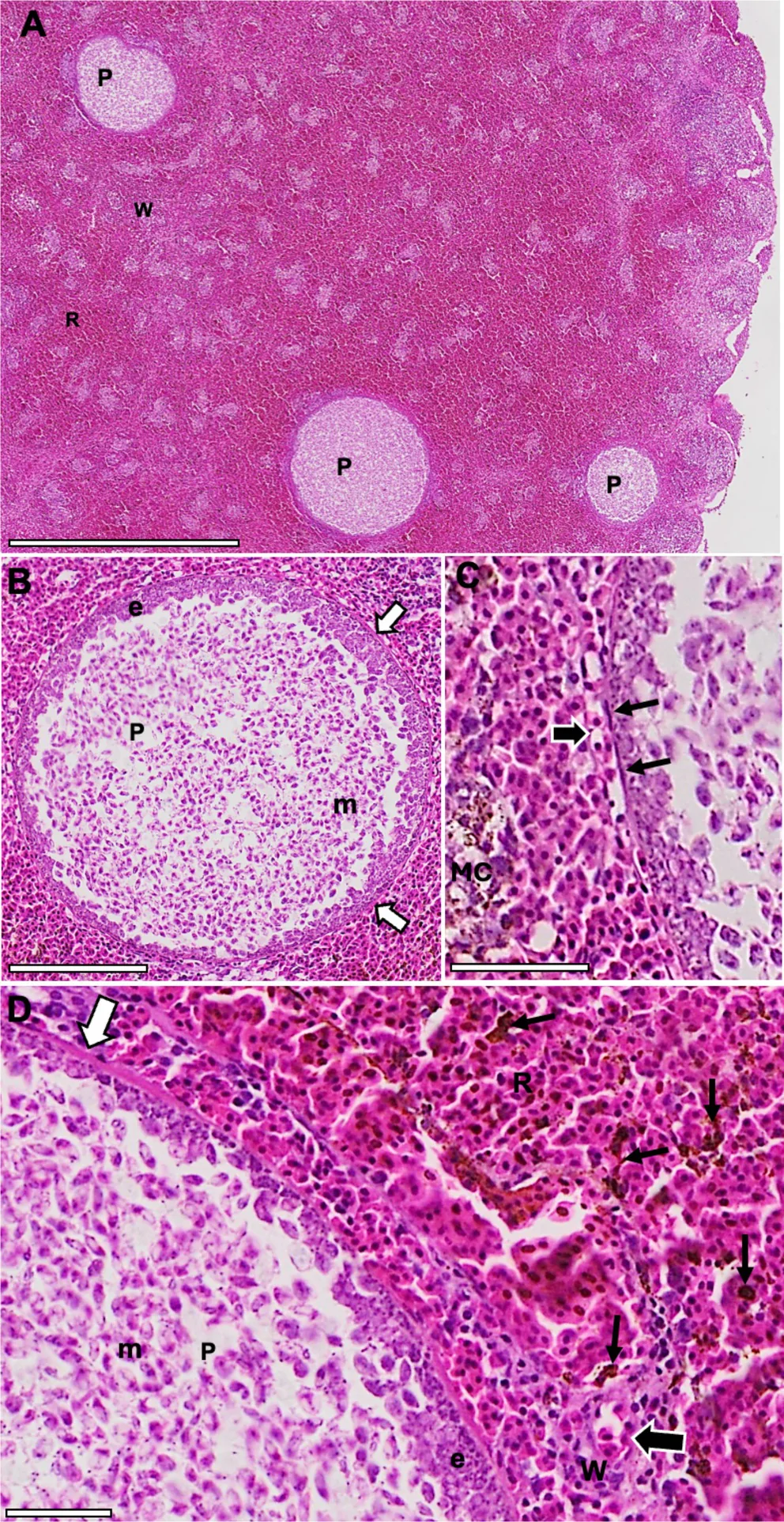
Photomicrographs of histological sections of the spleen of *Brycon hilarii* infected by *Myxobolus umidus*, HE stain. (A) Showing multiple large plasmodia (P) in distinct regions of the spleen. Note the white pulp (W) and red pulp (R). Scale bar = 1 mm. (B) Showing a plasmodium surrounded by a thin connective tissue capsule (large white arrow). Within the plasmodium (P), a peripheral layer of sporogonic developmental stages (e) and mature myxospores (m) in the deeper regions can be observed. Scale bar = 120 μm. (C) Showing the connective tissue capsule with elongated cell in an epithelioid arrangement (thin black arrows). A melanomacrophage center (MC) and a splenic capillary in longitudinal section (large black arrow) are also observed. Scale bar = 40 μm. (D) Edge of the plasmodium showing a connective tissue layer (large white arrow), a layer of sporogonic developmental stages (e) at the periphery of the plasmodium (P), and mature myxospores (m) in the deeper regions. Note the presence of dispersed melanomacrophages (thin black arrows) and an arteriole near the plasmodium (large black arrow). Scale bar = 40 μm.

The analysis of the gill filaments of *S. brasiliensis* infected by *M. pantanalis* revealed the presence of numerous plasmodia developing at the distal extremities of the filaments (Figure 3A,B). Plasmodial development occurs in the connective tissue or in the wall of blood vessels, occupying substantial portions of the filament tips (Figures 3 and 4). The infection induced important changes in the gill filaments, such as lamellar epithelial hyperplasia, lamellar fusion, lamellar edema, an evident inflammatory process, blood congestion, and focal necrosis of chondrocytes (Figures 3B, 4B and 5). The plasmodia were surrounded by a well‐defined layer of connective tissue (Figure 4B). Within the plasmodia, a thin peripheral layer of sporogonic developmental stages can be observed, whereas mature myxospores are localized in deeper regions (Figure 5A).

**FIGURE 3:**
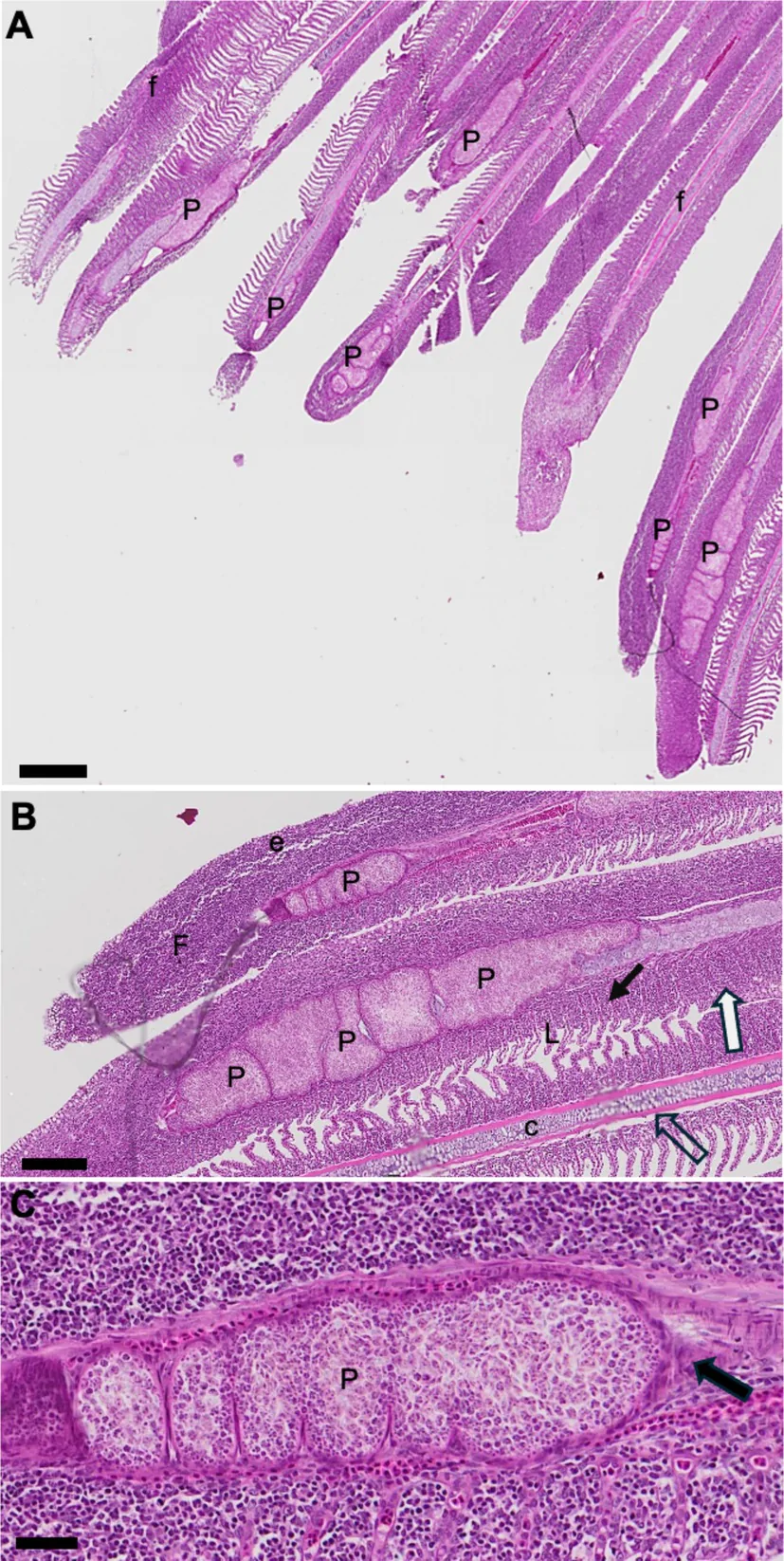
Photomicrographs of histological sections of the gill filaments of *Salminus brasiliensis* infected by *Myxobolus pantanalis*, HE stain. (A) Showing several large plasmodia (P) in the gill filaments (f). Scale bar = 0.5 mm. (B) Showing lamellar fusion (white arrow), hyperplasia of the lamellar epithelium (black arrow), and the perichondrium (empty arrow). Scale bar = 0.2 mm. (C) Amplified area of A showing the development of plasmodia (P) within the wall of an artery (note the muscular layer) (black arrow). Scale bar = 40 μm. c, cartilage; e, epithelium; F, Filament; L, lamellae.

**FIGURE 4:**
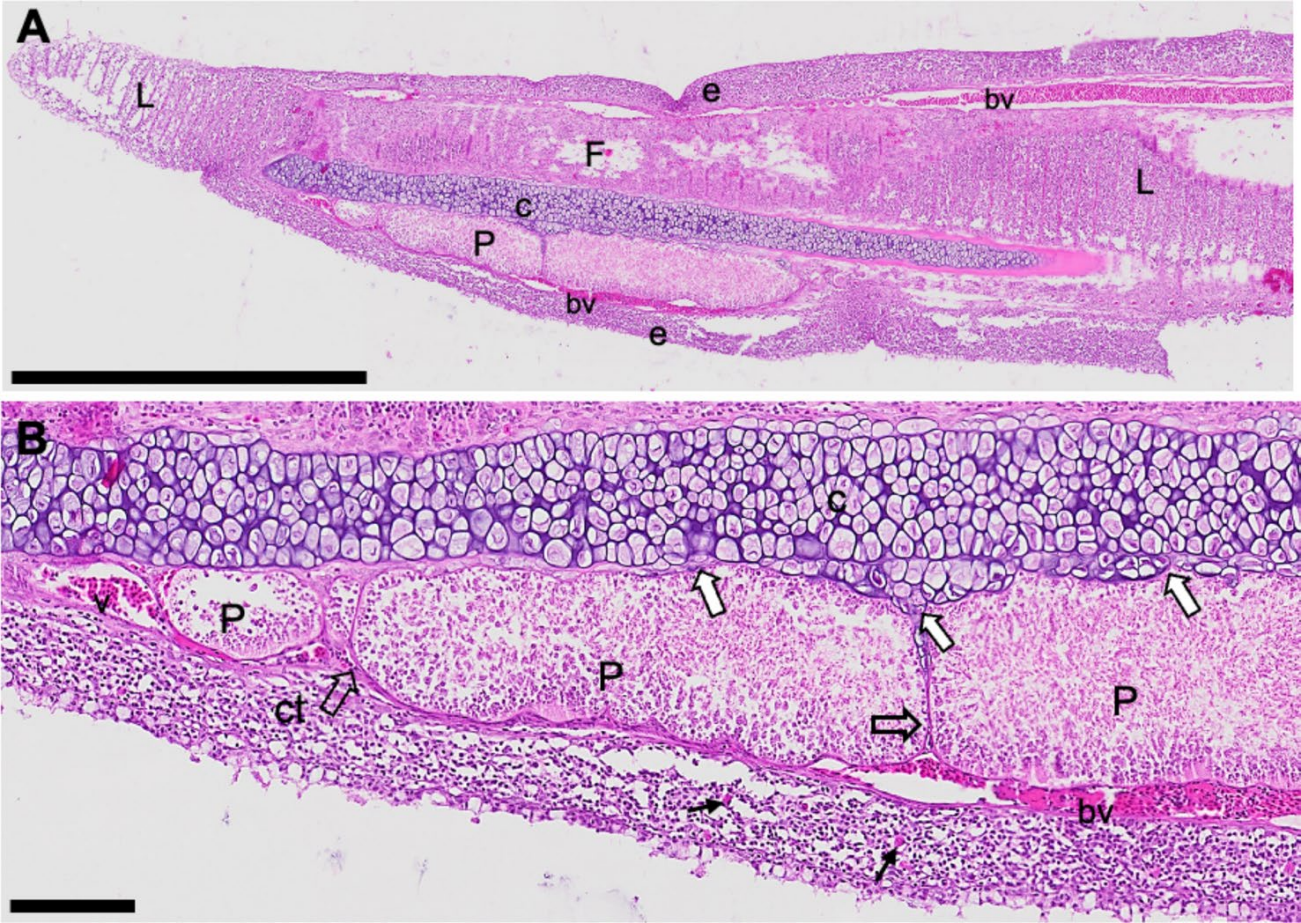
Photomicrograph of histological sections of the gill filament of *Salminus brasiliensis* infected by *Myxbolus pantanalis*. (A) Plasmodia (P) at the distal extremity of gill filaments (F). Scale bar: 1 mm. (B) Amplified area of A showing development of plasmodia (P) in the connective tissue (ct). Highlighting the proximity to the cartilaginous tissue (large white arrows) and blood vessels (v), the connective tissue layer surrounding the plasmodium (large empty arrows), and the presence of inflammatory cell infiltration (thin arrows). Scales bar: 100 μm. c, cartilage; e, epithelium; F, Filament; L, lamellae.

**FIGURE 5:**
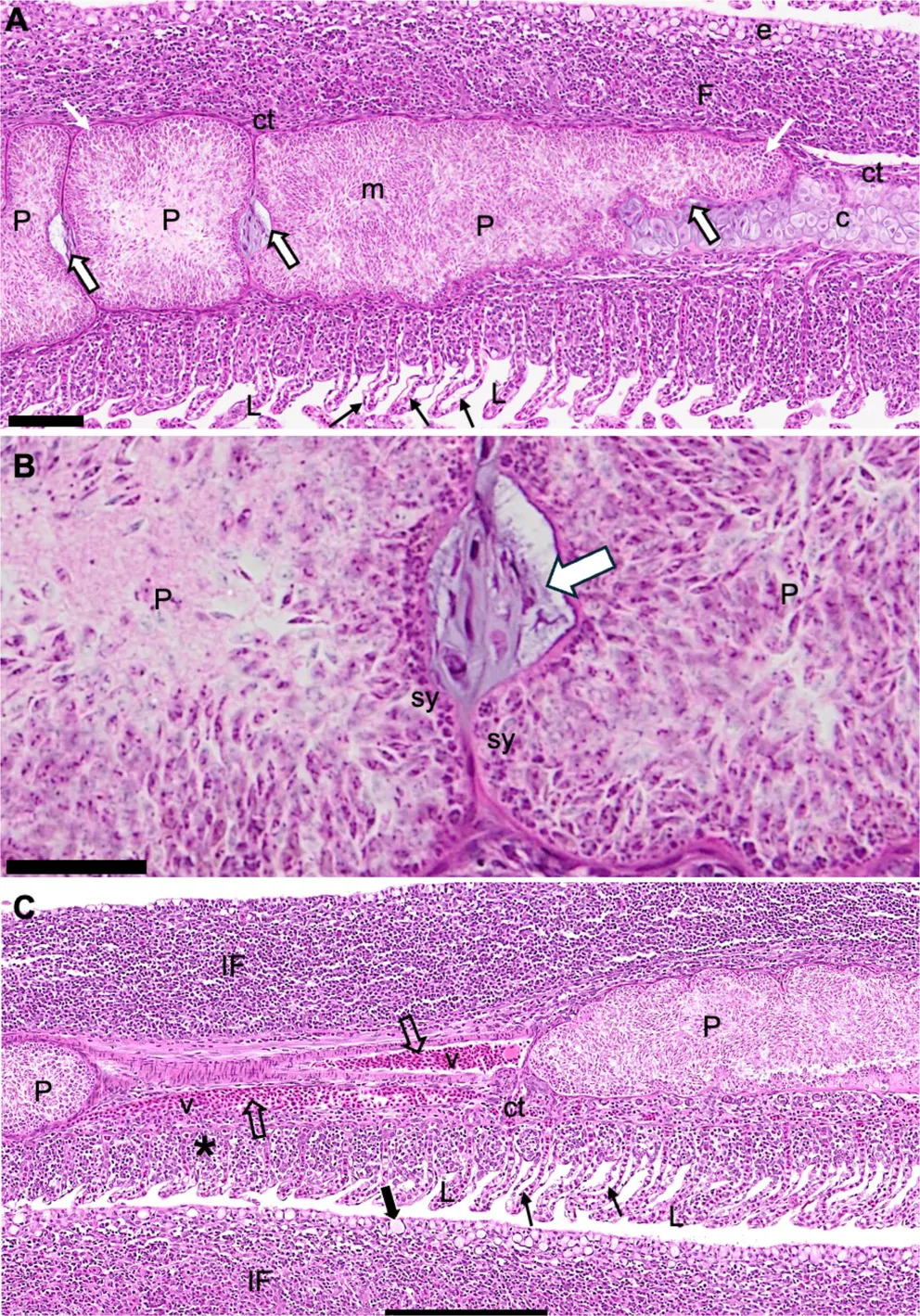
Photomicrograph of histological sections of the gill filament of *Salminus brasiliensis* infected by *Myxbolus pantanalis*. (A) Showing host–parasite interaction with the development of plasmodia (P) in the connective tissue (ct). Note the necroses of cartilaginous cells (large white arrows) and lamellar edema (thin black arrows). Within the plasmodium (P), a peripheral layer of sporogonic developmental stages (thin white arrows) and mature myxospores (m) in deeper regions can be observed. Scale bar: 100 μm. (B) Amplified area of A showing the area of necrosis of the cartilage tissue (large white arrow) between two plasmodia (P) and peripheral layer of sporogonic developmental stages (sy). Scale bar: 40 μm. (C) Showing presence of two plasmodia (P) and vessels (v) with blood congestion (large empty arrows). Note lamellar edema (thin black arrows) and areas with hyperplasia and lamellar fusion (asterisk), inflammatory cell infiltration (IF), and hyperplasia de globet cells (large black arrow). Scale bar: 200 μm. Cartilage (c), epithelium (e).

Partial SSU rDNA gene sequencing was performed for single samples of *M. umidus* and of *M. pantanalis*, yielding fragments of 1750 bp and 1915 bp, respectively. BLASTn search revealed 100% sequence identity with previously deposited sequences of these species (accession numbers KF296350 and KF296349), confirming the species identification. Additionally, the first SSU rDNA sequence of *M. salminus* was obtained from myxospores isolated from a single plasmodium, generating a 1812 bp fragment deposited in GenBank under accession number PX607271. BLASTn searches revealed that it did not match any other myxozoan sequence available in GenBank. The closest sequence identified was *Henneguya rotunda* Moreira et al. (2014) (query coverage 100.0%, maximum identities 91.8%), a parasite reported in the gill arch and fins of *S. brasilensis* from the Mogi Guaçu River, São Paulo state, Brazil.

Phylogenetic analysis placed *M. salminus* as a sister species to *H. rotunda* within a clade that also included *M. pantanalis* (Figure 6). Genetic distance analysis within this clade, based on SSU rDNA sequences, showed that *M. salminus* differed from *H. rotunda* by 7.9% (corresponding to 144 bp) and from *M. pantanalis* by 10.3% (corresponding to 184 bp).

**FIGURE 6:**
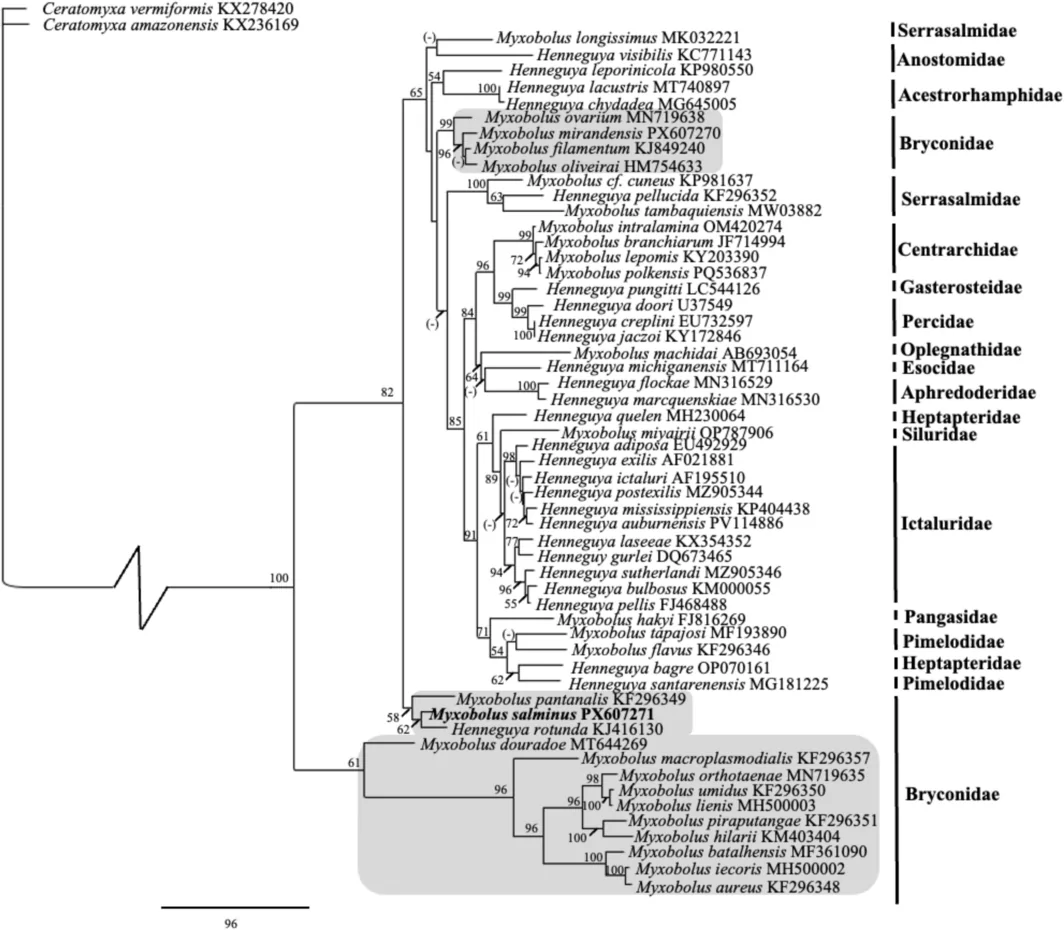
Maximum likelihood tree based on SSU rDNA sequences showing the phylogenetic positioning of *Myxobolus salminus*, parasite of the gill filaments of *Salminus brasiliensis* . Nodal supports are indicated for maximum likelihood (ML) with bootstrap at 1000 replicates. GenBank accession numbers are after the species name. Only branches with bootstrap support ≥ 50% are represented. Myxobolid parasites of bryconid fish are highlighted in grey.

## 4 Discussion

In the context of myxozoan taxonomy, a comprehensive approach that integrates multiple criteria has become increasingly crucial to ensure reliable species identification. Morphological characterization of the myxospore, together with host and tissue specificity and molecular data, provides a more robust framework for distinguishing taxa (Atkinson et al. 2015; Easy et al. 2005; Ferguson et al. 2008). This is particularly important for members of *Myxobolus*, whose congeners often share highly similar myxospore morphologies, and in some cases, exhibit morphological plasticity, as reported by Guo et al. (2018). However, the vast majority of *Myxobolus* species have been described primarily based on myxospore morphology (Eiras et al. 2021; Rocha et al. 2019).

Histological data are also fundamental in the study of histozoic myxozoan infections. This approach, in addition to allowing direct assessment of tissue tropism, which may be an important taxonomic character and a feature relevant to evolutionary analyses (Eszterbauer 2004; Shin et al. 2014), is also crucial for identifying tissue damage resulting from infection (Figueredo et al. 2023; Holzer et al. 2021; Woodyard et al. 2022). With the aim of expanding available knowledge derived from integrative analyses of myxosporean parasites of commercially important South American fish species, we provide SSU rDNA sequence data for *M. salminus*, a parasite of the gills of *S. brasiliensis* , whose original description was based on myxospore morphology and histological and ultrastructural observations (Adriano et al. 2009). In addition, we present histological data on infections of the spleen of *B. hilarii* by *M. umidus* and of the gills of *S. brasiliensis* by *M. pantanalis*, both of which were originally described using morphological and SSU rDNA data (Carriero et al. 2013).

With regard to *M. salminus*, the SSU rDNA sequence obtained, in addition to providing genetic data useful for a more confident species differentiation, allowed us to infer its phylogenetic position, which revealed it grouping within a lineage composed of three other parasite species of *S. brasiliensis* ( *H. rotunda* and *M. pantanalis*). This finding corroborates previous reports indicating a strong tendency for species of the *Myxobolus* lato sensu group to cluster according to the phylogenetic relationships of their fish hosts (Carriero et al. 2013; Fiala 2006; Liu et al. 2019). However, our analyses further revealed that *Myxobolus/Henneguya* species infecting bryconid do not constitute a monophyletic lineage, a condition likewise reported for other fish families (Carriero et al. 2013; Moreira et al. 2014; Liu et al. 2019).

Aiming to fill the gap in knowledge regarding host–parasite interactions for *M. umidus* infecting the spleen of *B. hilarii* and *M. pantanalis* infecting the gill filaments of *S. brasiliensis* , we provide here histopathological data for these species. The development of several large plasmodia of *M. umidus* in the spleen, which may reach up to 0.8 mm, was not associated with an inflammatory response, and the white and red pulp retained the typical structures and general organization, as described to spleen in teleost (Zapata 2024). Melanomacrophage were present throughout the organ as expected, but they did not contain parasites (Figure 2C), despite their common association with responses to microorganisms, including myxozoans (Agius and Roberts 2003; Sitjà‐Bobadilla 2008; Woo 2006). The plasmodia were separated from the host tissue by a tenuous capsule of connective tissue, characterized by elongated cells in an epithelioid arrangement, which has also been commonly observed in myxozoan infections (Sitjà‐Bobadilla 2008). Histopathological studies of splenic myxozoan infections are scarce in the literature and have largely been restricted to describing effects associated just with myxospore presence. Myxospores of *Myxobolus xiaoganensis* Zhang et al. 2023, reported infecting the spleen of silver carp, were described within melanomacrophage centers and surrounded by hemosiderin pigments, indicating active splenic sequestration and processing (Zhang et al. 2023). Similar patterns have been described in experimental infections with *Enteromyxum scophthalmi* Palenzuela, Redondo & Alvarez‐Pellitero, 2002 in turbot, in which increased macrophage abundance and occasional intracellular parasite stages were observed in the spleen without evident impairment of haematopoiesis (Sitjà‐Bobadilla et al. 2006). Thus, our histopathological findings provide insights into the development of myxozoan plasmodia within the spleen, which occurs without eliciting an inflammatory response. This is possibly due to the encapsulation of plasmodia, a phenomenon that represents a form of evolutionary adaptation and immune evasion, in which the connective tissue envelope acts as a physical barrier, preventing direct contact between immune cells and the parasite (Holzer et al. 2021; Sitjà‐Bobadilla 2008).

In the case of *M. pantanalis*, plasmodial development occurred in the connective tissue of the gill filaments, occupying substantial portions of the filament tips. The infection caused notable damage to adjacent tissues and structures, including inflammatory process, pronounced compression of the connective tissue and cartilaginous matrix, focal necrosis of chondrocytes, lamellar fusion and lamellar hyperplasia, and vascular alterations such as blood congestion and lamellar edema. As the gill epithelium constitutes the first barrier to infection, the innate immune system is rapidly activated, primarily through mucosal defences (Holzer et al. 2021). This response explains the frequent occurrence of epithelial hyperplasia in the lamellae, particularly involving goblet cells, as observed in the infections by *M. pantanalis*. With the development of the infection, additional immune mechanisms are triggered, resulting in the inflammatory process observed. This scenario highlights the significant pathogenic potential of this myxozoan species in a fish host of considerable importance for commercial fisheries, sport fishing, and aquaculture (Gimênes Junior and Rech 2022; Resende 2003; Vitti et al. 2013). Comparable alterations have been documented in other myxozoan infections of fish gills (Meira et al. 2025; Molnár et al. 2006; Naldoni et al. 2009), and also indicates that, in addition to epithelial involvement and the inflammatory process, pathological importance may be attributed to the possible mechanical pressure exerted by histozoic myxozoan plasmodial development (Feist and Longshaw 2006).

In conclusion, this study provides a valuable contribution to the characterization of three *Myxobolus* species infecting South American bryconid fishes by presenting the histological characterization of infections caused by *M. umidus* and *M. pantanalis*, as well as the sequencing of the SSU rDNA of *M. salminus* and its phylogenetic positioning. Together, these data expand the available dataset for taxonomic studies and host–parasite relationship analyses involving parasites that infect economically important South American freshwater fishes.

## Author Contributions

**Caroline M. Meira:** writing – original draft, visualization, validation, software, methodology, investigation, formal analysis, data curation, conceptualization, writing – review and editing. **Milena L. Cornejo:** methodology, formal analysis. **Sarah Arana:** writing – review and editing, validation, data curation. **Edson A. Adriano:** writing – review and editing, validation, resources, investigation, funding acquisition, data curation, conceptualization. **Antonio A. M. Maia:** writing – review and editing, supervision, resources, funding acquisition.

## Funding

This study was funded by the São Paulo Research Foundation ‐ FAPESP (grant #2018/24980‐8). C. M. Meira was supported with a PhD. scholarship provided by FAPESP (grant #2023/15107‐7) and by the Conselho Nacional de Desenvolvimento Científico e Tecnológico – CNPq/Brasil (grant #140004/2023‐8). M. L. Cornejo received a grant from the Progama Unificado de Bolsas da Universidade de São Paulo – PUB/USP. E. A. Adriano received a research productivity grant from the CNPq (grant #307485/2023‐4). This study was financed in part by the Coordenação de Aperfeiçoamento de Pessoal de Nível Superior – Brasil (CAPES) – Finance Code 001.

## Conflicts of Interest

The authors declare no conflicts of interest.

## Data Availability

All data generated or analysed during this study are included in the article. The sequence obtained in this study has been deposited in GenBank under accession number PX607271.
